# Reduced serum vitamin D levels are associated with poor sleep quality in early stroke patients

**DOI:** 10.3389/fnut.2022.937867

**Published:** 2022-07-22

**Authors:** Guiqian Huang, Jiahao Chen, Luqian Zhan, Jingfang Long, Yisi Lin, Beilei Zhu, Jincai He

**Affiliations:** Department of Neurology, The First Affiliated Hospital of Wenzhou Medical University, Wenzhou, China

**Keywords:** acute ischemic stroke, sleep quality, depression, vitamin D, 25(OH)D

## Abstract

**Background:**

Poor sleep quality and vitamin D deficiency are common in stroke patients. Our aim was to evaluate the possible association between vitamin D and sleep quality in acute ischemic stroke (AIS) patients.

**Methods:**

A total of 301 AIS patients were screened and completed 1-month follow-up. Serum 25-hydroxyvitamin D [25(OH)D] was used to assess the vitamin D status by a competitive protein-binding assay at baseline. All patients were divided into equal quartile according to the distribution of 25(OH)D. One month after stroke, sleep quality was evaluated by using Pittsburgh Sleep Quality Index (PSQI) and Epworth Sleepiness Scale (ESS) questionnaire; depression status was confirmed by 17-item Hamilton Depression Scale (HAMD).

**Results:**

There were 89 (29.6%) AIS patients with poor sleep quality 1-month post-event. Within 24 h after admission, serum 25(OH)D levels were significantly lower in patients with poor sleep quality after stroke (*P* < 0.001). In the results of multivariate-adjusted logistic regression analysis, the odds ratio (OR) of poor sleep quality was 6.199 (95% CI, 2.066–18.600) for the lowest quartile of 25(OH)D compared with the highest quartile. In patients without depression, reduced 25(OH)D were still significantly associated with poor sleep quality (OR = 8.174, 95% CI = 2.432–27.473). Furthermore, 25(OH)D and HAMD score were combined to enhance the diagnostic accuracy of poor sleep quality, with the area under the receiver operating characteristic curve of 0.775.

**Conclusion:**

Reduced serum levels of vitamin D at admission were independently and significantly associated with poor sleep quality at 1 month after stroke. Our findings suggested the combination of vitamin D and depression status could provide important predictive information for post-stroke sleep quality.

## Introduction

Stroke patients often experienced sleep problems such as poor sleep quality, difficulty in falling asleep, daytime sleepiness, and sleep-disordered breathing (1, 2). Importantly, as previously reported, sleep quality was found to be associated with cardiovascular disease, diabetes, obesity, negative psychological consequences and neurocognitive dysfunctions (3–5). In stroke patients, poor quality specifically affects daytime functional outcomes including mobility, fatigue, and recovery outcomes (6, 7). It may also affect patients’ participation in therapies (8). Overall, poor sleep quality were important causes of poor prognosis in stroke survivors (9).

In spite of this, the preponderance of research in stroke primarily focus on obstructive sleep-disordered breathing rather than sleep quality (10, 11). Furthermore, the underlying pathophysiology of sleep quality after stroke remains unclear yet. Recent studies have shown that low serum 25-hydroxyvitamin D [25(OH)D] levels were related to neuropsychiatric diseases and effects on brain development (12). Especially, vitamin D plays a significant role in the regulation of sleep (13). People with vitamin D deficiency were prone to difficulty sleeping, trouble maintaining asleep, shorter sleep duration, and nocturnal awakenings (14, 15). And a recent study found sleep quality can be improved with vitamin D supplementation (16). But this relationship is still controversial (17).

The association between the vitamin D and sleep has been observed in several healthy and ill populations, including elderly in the community, pregnant women and factory workers (13). However, to our knowledge, this association has not been investigated in stroke patients, whose prevalence of vitamin D deficiency was high (30–77%) (18, 19). Therefore, we would like to know whether such relationships exist in the stroke population. The primary aim of this study is to explore the relationship between serum vitamin D levels and the underlying risk of post-stroke sleep quality deterioration, and further explore whether serum vitamin D can predict sleep quality in early stroke patients.

## Materials and methods

### Study design

This is a prospective and comparative observational study of consecutive patients with acute ischemic stroke (AIS) enrolled in the First Affiliated Hospital of Wenzhou Medical University between May 2014 and March 2018.

Written informed consents were signed voluntarily by all subjects or their immediate family members. The study was conducted in accordance with the Declaration of Helsinki and was approved by the Medical Ethics Committee of the First Affiliated Hospital of Wenzhou Medical University.

### Inclusion and exclusion criteria

All patients with suspected AIS were admitted to the hospital within 1 week of onset, and AIS was confirmed by magnetic resonance imaging (MRI) or computed tomography (CT) within 72 h of hospitalization. The exclusion criteria were as follows: (i) missing vitamin D records, (ii) diagnosed with transient ischemic attacks, (iii) history of respiratory disorders, such as chronic obstructive pulmonary disease and chronic cough, (iv) previously diagnosed with sleep disorder under treatment, (v) night shift work or abnormal sleep schedules, (vi) osteoporosis or vitamin D supplementation before stroke onset, (vii) any central nervous system disease, such as dementia or Parkinson’s disease, (viii) a history of major depression or other psychiatric disorders, (ix) severe aphasia, dysarthria, or cognitive impairment preventing complete psychological assessments, and (x) lost to follow-up. Ultimately, the study included 301 eligible patients.

### Data collection and follow-up

The patients’ medical records were reviewed, and demographic information, including age, body mass index (BMI), education years, and marital status, was collected. All patients underwent a baseline clinical evaluation and an assessment of vascular risk factors, including current smoking status, current alcohol consumption status, and histories of stroke, dyslipidemia, hypertension, diabetes, and coronary artery disease (CAD). Clinical and laboratory data within 1 day of admission were obtained from electronic medical records. All subjects underwent cranial CT or MRI to confirm the AIS lesion location within 72 h after admission. Experienced neurologists assessed stroke severity within 24 h of admission according to the National Institutes of Health Stroke Scale (NIHSS). The NIHSS score ranges from 0 to 42, with higher scores indicating more severe neurological deficits. Functional status was estimated using the Barthel Index (BI) and modified Rankin Scale (mRS) at hospital discharge. Patients were followed 1 month after acute stroke.

### Vitamin D measurement

The serum 25(OH)D level was used to assess the vitamin D status of all participants, and was tested within 24 h of hospitalization (at the baseline). The serum 25(OH)D level was measured using a competitive electrochemiluminescence protein binding assay (COBAS e602; Roche Diagnostics, Germany) in our hospital’s biochemistry department. The intra-assay coefficient of variation was 7–10%.

According to the distribution of 25(OH)D levels, the 25(OH)D levels were further stratified into quartiles to determine whether enhanced performance could be quantified with sufficient statistical power.

### Sleep quality measurement

Although polysomnography is the gold standard for diagnosing sleep problems, it cannot be used in all stroke patients with physical disability because of its expense and limited accessibility. And because of the high variability of insomniac sleep and bias related to the first night effect, polysomnography is not indicated for routine evaluation of chronic sleep trouble (20). Therefore, additional tools, such as valid sleep questionnaires, are needed to evaluate more stroke patients. In our study, sleep quality was assessed by trained neurologists using the Chinese version of Pittsburgh Sleep Quality Index (PSQI) during the outpatient follow-up of 1 month after stroke onset (21), which is the most frequently used measures in studies collecting prevalence data (22). The PSQI comprises 19 self-reported questions among the following seven component scales, which are based on sleep in the past month: sleep quality, sleep duration, sleep latency, sleep disturbances, sleep efficiency, use of sleep medication, and daytime dysfunction. Each response is scored from 0 to 3, where 0 indicates “no symptoms,” 1 indicates “once a week,” 2 indicates “once or twice a week,” and 3 indicates “three or more times per week.” The sum of the scores yields the global sleep quality score with a range of 0–21. Higher scores (>7) indicate worse sleep quality (21).

We also used the Epworth Sleepiness Scale (ESS) (23) to assess daytime sleepiness 1 month after the onset of stroke, as another measurement of sleep quality. This questionnaire is composed of eight questions evaluating the tendency of falling asleep or dozing off under eight different scenarios over the previous month. The score of each item ranges from 0 to 3 (0 represents never dozing, 1 a slight chance of dozing, 2 a moderate chance of dozing, and 3 a high chance of dozing); on summation, the total score ranges from 0 to 24. To ensure that the scores are comparable in our population, threshold values taken from previous studies (24, 25) were used to classify daytime dozing into three grades: none (total score = 0), mild/moderate (1–9), and severe (≥10).

### Psychological measurements

Depression symptoms were measured using the 17-item Hamilton Depression Scale (HAMD) (26) at 1 month after the onset of stroke. Patients with a HAMD score >7 were diagnosed according to the Structured Clinical Interview of the Diagnostic and Statistical Manual of Mental Disorders, fourth edition. The mental and sleep condition evaluations were performed by experienced neurologists blinded to the results of the other clinical and laboratory examinations.

### Statistical analysis

The Kolmogorov–Smirnov test was performed to determine whether the variable distributions were normal or not. Based on the distribution type, continuous variables are presented as mean ± SD or as the median with the interquartile range (IQR). Categorical variables are presented as the relative frequency with percentage. Student’s *t*-test and the Mann–Whitney U-test were used to compare continuous variables, as appropriate. The Chi-square test was used to compare categorical variables. Statistical comparisons among vitamin D levels were analyzed using one-way analysis of variance (ANOVA) or the Kruskal–Wallis test for continuous variables and Pearson’s Chi-square test or Fisher’s exact test for categorical variables. Univariate analysis was used to identify significant variables (*P* < 0.1) associated with the risk of poor sleep quality. These significant variables and conventional confounding factors (27–29) were selected as covariates to enter multivariate-adjusted binary logistic regression models, which were used to identify independent risk factors for poor sleep quality after AIS. Furthermore, we performed tests for linear trend by entering the median value of each category of serum 25(OH)D as a continuous variable in the models. A receiver operating characteristic (ROC) curve analysis was used to determine the diagnostic accuracy of poor sleep quality after AIS. The area under the ROC curve (AUROC) is presented with 95% CIs and was compared between the 25(OH)D and other models using the method of Hanley and McNeil. In this study, statistical significance was defined as a two-tailed *P* < 0.05. All analyses were performed using SAS ver. 9.2 (SAS Institute, Cary, NC, United States) or MedCalc ver. 12.7 (MedCalc Software, Ostend, Belgium).

## Results

### Baseline characteristics of the patients according to sleep quality

**FIGURE 1:**
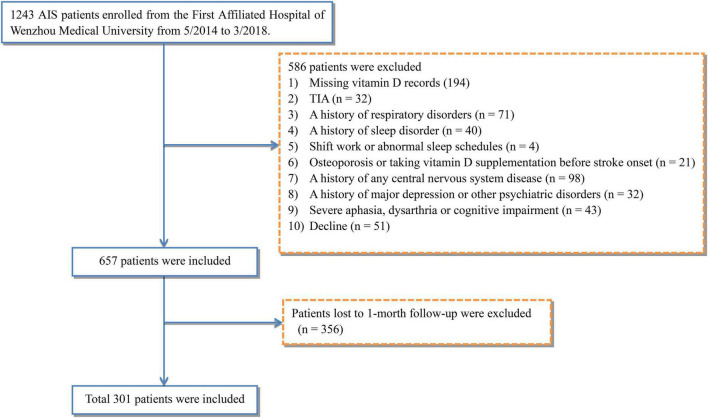
Study flow diagram. AIS, acute ischemic stroke; TIA, transient ischemic attacks.

This study enrolled 1,243 AIS patients from the First Affiliated Hospital of Wenzhou Medical University between May 2014 and March 2018. Among them, 657 eligible patients were included according to exclusion criteria. However, due to 356 patients’ loss to follow-up, a total of 301 patients (187 males and 114 females, with mean age at 62.2 years) were consequently included in this study (Figure 1).

**FIGURE 2:**
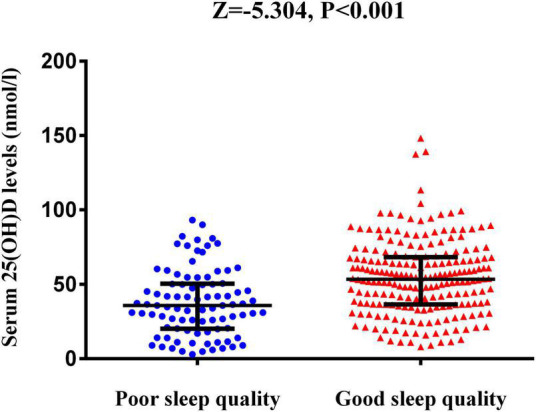
The serum 25(OH)D levels between AIS patients with poor sleep quality and those with good sleep quality. The horizontal lines indicate median levels and interquartile ranges (IQRs). *P*-values refer to Mann–Whitney U tests for differences between groups. 25(OH)D, 25-hydroxyvitamin D.

Table 1 shows the baseline characteristics of the patients with good sleep quality and poor sleep quality after AIS. Good sleep quality was defined as a PSQI score ≤7 at 1 month after the onset of stroke and poor sleep quality as a PSQI score >7. In this study, 89 (29.6%) patients had poor sleep quality at the 1-month follow-up. Compared with good sleep quality, patients with poor sleep quality had higher NIHSS, mRS, and HAMD scores (Table 1). The serum 25(OH)D level was significantly lower in patients with poor sleep quality versus good sleep quality [35.7 nmol/L (IQR, 20.3–50.2) vs. 53.4 nmol/L (IQR, 36.5–68.0); *P* < 0.001; Figure 2].

**TABLE 1:** Clinical and demographic characteristics of AIS patients, stratified by sleep quality.

Variables	Good sleep quality (*n* = 212)	Poor sleep quality (*n* = 89)	*P*-value
Laboratory parameters			
25(OH)D (nmol/L)	53.4 (36.5–68.0)	35.7 (20.3–50.2)	<0.001
White blood cell (10^9^/L)	6.8 ± 2.0	6.9 ± 1.9	0.737
Red blood cell (10^9^/L)	4.5 ± 0.6	4.5 ± 0.6	0.854
Hemoglobin (g/L)	136.5 ± 18.8	136.7 ± 17.7	0.926
Platelet (10^9^/L)	206.2 ± 49.2	219.0 ± 58.6	0.053
ALT (IU/L)	18.0 (13.0–26.0)	17.0 (12.0–22.0)	0.313
AST (IU/L)	21.0 (17.0–26.0)	19.0 (16.0–25.0)	0.096
Blood urea nitrogen (mmol/L)	4.8 (3.9–5.8)	5.2 (3.8–5.9)	0.811
SCr (μmol/L)	70.0 (59.0–85.0)	71.0 (60.0–88.0)	0.542
Uric acid (μmol/L)	289.2 ± 89.7	293.9 ± 89.6	0.678
Fast blood glucose (mmol/L)	5.0 (4.5–6.4)	5.0 (4.6–6.2)	0.911
HDL (mmol/L)	1.1 (0.9–1.2)	1.0 (0.9–1.2)	0.319
LDL (mmol/L)	2.8 ± 0.9	2.9 ± 0.9	0.265
Prealbumin (g/L)	262.0 ± 79.0	248.7 ± 83.4	0.234
Vitamin B12 (pg/ml)	350.5 (245.8–456.2)	337.0 (254.0–437.0)	0.700
Demographic characteristics			
Age (years)	62.1 ± 10.1	62.3 ± 11.4	0.871
Gender, male, *n* (%)	134 (63.2%)	53 (59.6%)	0.551
BMI (kg/m^2^)	24.4 ± 3.1	24.5 ± 3.2	0.679
Education years (years)	4.1 ± 3.8	4.0 ± 3.5	0.938
Marital status, married, *n* (%)	196 (92.5%)	78 (87.6%)	0.190
Vascular risk factors, *n* (%)			
History of hypertension, *n* (%)	154 (73.3%)	59 (67.0%)	0.273
History of diabetes, *n* (%)	53 (25.4%)	20 (22.7%)	0.631
History of hyperlipidemia, *n* (%)	18 (8.6%)	9 (10.2%)	0.662
History of previous stroke, *n* (%)	21 (10.0%)	9 (10.2%)	0.963
History of coronary artery disease, *n* (%)	13 (6.2%)	7 (8.0%)	0.613
Current smoking, *n* (%)	103 (49.3%)	38 (44.2%)	0.426
Current drinking, *n* (%)	71 (35.7%)	26 (31.3%)	0.483
Lesion location			
Frontal lobe, *n* (%)	32 (23.7%)	9 (18.0%)	0.407
Parietal lobe, *n* (%)	15 (11.5%)	4 (8.3%)	0.785
Temporal lobe, *n* (%)	11 (8.6%)	6 (12.0%)	0.571
Occipital lobe, *n* (%)	13 (10.1%)	7 (13.7%)	0.599
Basal ganglia, *n* (%)	94 (60.6%)	41 (60.3%)	0.961
Cerebellum, *n* (%)	19 (14.3%)	8 (16.0%)	0.816
Midbrain, *n* (%)	23 (17.3%)	10 (20.8%)	0.663
Pons, *n* (%)	33 (23.7%)	15 (30.6%)	0.342
Medulla, *n* (%)	23 (17.3%)	11 (22.4%)	0.520
Clinical characteristics			
NIHSS score, median (IQR)	2.0 (1.0–4.0)	3.0 (2.0–4.0)	0.031
BI score, median (IQR)	100.0 (83.8–100.0)	95.0 (80.0–100.0)	0.087
mRS score, median (IQR)	1.0 (1.0–3.0)	2.0 (1.0–3.0)	0.023
HAMD score, median (IQR)	4.0 (2.0–6.0)	7.0 (4.0–10.0)	<0.001

AIS, acute ischemic stroke; ALT, alanine aminotransferase; AST, aspartate aminotransferase; BMI, body mass index; BI, Barthel Index; HAMD, Hamilton Depression Scale; HDL, high-density-lipoprotein; LDL, low-density-lipoprotein; mRS, modified Rankin Scale; NIHSS, National Institutes of Health Stroke Scale; SCr, serum creatinine concentration; 25(OH)D, 25-hydroxyvitamin D.

### Baseline characteristics of the patients according to serum 25-hydroxyvitamin D quartile

**FIGURE 3:**
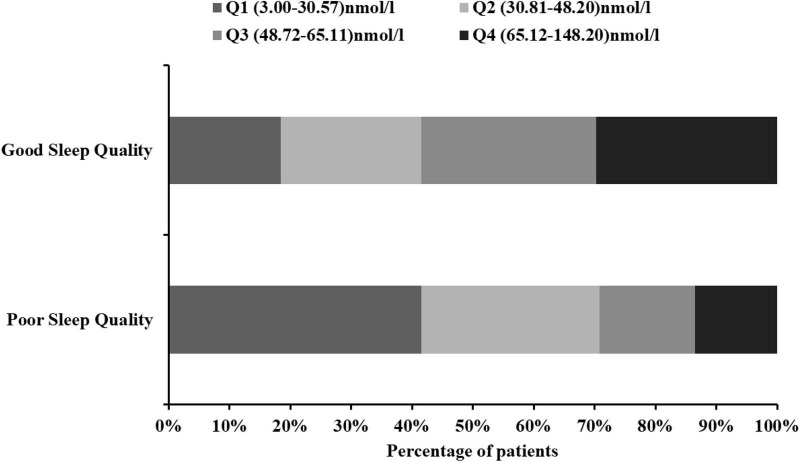
Proportion of people between 25(OH)D quartiles in AIS patients with different sleep quality. 25(OH)D, 25-hydroxyvitamin D.

The patients were divided into four subgroups according to quartiles of the serum 25(OH)D levels, which ensured that per subgroups contained adequate number of patients from the range of 3.00 to 148.20 nmol/L (Q1, 76 patients; Q2, 75 patients; Q3, 75 patients; and Q4 75 patients). The cut-off values for this stratification of the 25(OH)D were: (Q1) 3.00–30.57 nmol/L, (Q2) 30.81–48.20 nmol/L, (Q3) 48.72–65.11 nmol/L, and (Q4) 65.12–148.20 nmol/L. In Table 2, we summarized the demographic, imaging, laboratory, and clinical characteristics of the AIS patients by 25(OH)D quartile. The occipital lobe, history of hypertension, history of diabetes, and current drinking status among the patients with different quartiles of serum 25(OH)D were significantly different (all *P* < 0.05; Table 2). The lower 25(OH)D quartile was significantly associated with high PSQI score, Epworth score, and HAMD score (*P* = 0.002, *P* = 0.037, *P* = 0.047, respectively). The proportion of patients in Q1 [3.00–30.57 nmol/L 25(OH)D] was significantly lower in the good sleep quality group (*P* = 0.004; Figure 3), whereas the proportion of patients in Q4 [65.12–148.20 nmol/L 25(OH)D] was significantly higher in the good sleep quality group (*P* < 0.001; Figure 3). The other baseline characteristics did not differ significantly according to the 25(OH)D quartile.

**TABLE 2:** Baseline characteristics of AIS patients in 25(OH)D quartiles.

Variables	All patients	25(OH)D quartiles				
		
		Quartile 1 *n* = 76 (3.00–30.57)	Quartile 2 *n* = 75 (30.81–48.20)	Quartile 3 *n* = 75 (48.72–65.11)	Quartile 4 *n* = 75 (65.12–148.20)	*P*-value
25(OH)D (nmol/L)	48.2 (30.6–65.1)	19.5 (11.3–26.1)	39.0 (36.0–43.5)	56.6 (52.1–60.1)	79.9 (71.8–88.0)	<0.001
Demographic parameters						
Age (years)	62.2 ± 10.5	60.9 ± 11.1	61.4 ± 10.6	64.1 ± 9.0	62.2 ± 11.0	0.275
Gender						0.099
Male, *n* (%)	187 (62.1%)	40 (52.6%)	44 (58.7%)	50 (66.7%)	53 (70.7%)	
Female, *n* (%)	114 (37.9%)	36 (47.4%)	31 (41.3%)	25 (33.3%)	22 (29.3%)	
BMI (kg/m^2^)	24.4 ± 3.2	24.7 ± 3.6	24.5 ± 3.2	24.2 ± 2.9	24.1 ± 2.9	0.628
Education (years)	4.1 ± 3.7	4.8 ± 3.9	4.1 ± 4.1	4.2 ± 3.4	3.2 ± 3.4	0.061
Marital status						
Married, *n* (%)	274 (91.0%)	65 (85.5%)	67 (89.3%)	72 (96.0%)	70 (93.3%)	0.120
Lesion location						
Frontal lobe, *n* (%)	41 (22.2%)	12 (23.1%)	9 (20.5%)	13 (27.7%)	7 (16.7%)	0.646
Parietal lobe, *n* (%)	19 (10.6%)	5 (10.2%)	2 (4.9%)	8 (17.0%)	4 (9.5%)	0.319
Temporal lobe, *n* (%)	17 (9.6%)	5 (10.2%)	4 (9.8%)	7 (14.6%)	1 (2.5%)	0.291
Occipital lobe, *n* (%)	20 (11.1%)	3 (6.2%)	4 (9.5%)	12 (24.0%)	1 (2.5%)	0.005
Basal ganglia, *n* (%)	135 (60.5%)	36 (59.0%)	32 (60.4%)	34 (58.6%)	33 (64.7%)	0.915
Cerebellum, *n* (%)	27 (14.8%)	6 (12.8%)	5 (11.6%)	10 (20.4%)	6 (13.6%)	0.621
Midbrain, *n* (%)	33 (18.2%)	7 (14.9%)	6 (14.3%)	10 (21.3%)	10 (22.2%)	0.668
Pons, *n* (%)	47 (25.5%)	12 (25.0%)	9 (21.4%)	12 (25.0%)	14 (30.4%)	0.809
Medulla, *n* (%)	34 (18.7%)	7 (14.9%)	7 (16.3%)	10 (21.3%)	10 (22.2%)	0.756
Vascular risk factors						
History of hypertension, *n* (%)	213 (71.5%)	49 (64.5%)	64 (85.3%)	49 (66.2%)	51 (69.9%)	0.019
History of diabetes, *n* (%)	73 (24.6%)	18 (23.7%)	23 (30.7%)	24 (32.4%)	8 (11.1%)	0.012
History of coronary artery disease, *n* (%)	20 (6.8%)	4 (5.3%)	2 (2.7%)	9 (12.2%)	5 (6.9%)	0.131
History of hyperlipidemia, *n* (%)	27 (9.1%)	11 (14.5%)	5 (6.7%)	7 (9.5%)	4 (5.6%)	0.231
History of a previous stroke, *n* (%)	30 (10.1%)	10 (13.2%)	9 (12.0%)	5 (6.8%)	6 (8.3%)	0.524
Current smoking, *n* (%)	141 (47.8%)	30 (41.1%)	35 (46.7%)	36 (48.6%)	40 (54.8%)	0.422
Current drinking, *n* (%)	97 (34.4%)	15 (21.1%)	23 (31.1%)	26 (37.1%)	33 (49.3%)	0.005
Clinical characteristics						
NIHSS score, median (IQR)	2.0 (1.0–4.0)	2.0 (1.0–4.0)	2.0 (1.0–4.0)	2.5 (1.0–4.0)	3.0 (1.0–4.0)	0.999
BI score, median (IQR)	100.0 (80.0–100.0)	95.0 (77.5–100.0)	100.0 (85.0–100.0)	100.0 (85.0–100.0)	100.0 (75.0–100.0)	0.955
mRS score, median (IQR)	1.0 (1.0–3.0)	2.0 (1.0–3.0)	1.0 (1.0–2.0)	1.0 (1.0–3.0)	1.0 (1.0–3.0)	0.506
PSQI score, median (IQR)	5.0 (3.0–8.0)	7.0 (4.0–10.2)	5.0 (3.0–9.0)	5.0 (3.0–7.0)	4.0 (3.0–6.0)	0.002
Epworth score						0.037
0	47 (16.0%)	8 (10.5%)	11 (15.0%)	12 (16.2%)	16 (22.5%)	
1–9	179 (60.9%)	41 (54.0%)	44 (60.3%)	48 (64.9%)	46 (64.8%)	
>9	68 (23.1%)	27 (35.5%)	18 (24.7%)	14 (18.9%)	9 (12.7%)	
HAMD score, median (IQR)	5.0 (2.0–7.8)	5.0 (2.0–9.0)	6.0 (3.0–8.8)	4.0 (2.5–6.5)	4.0 (1.0–7.0)	0.047

AIS, acute ischemic stroke; BMI, body mass index; BI, Barthel Index; HAMD, Hamilton Depression Scale; IQR, interquartile range; mRS, modified Rankin Scale; NIHSS, National Institutes of Health Stroke Scale; PSQI, Pittsburgh Sleep Quality Index; 25(OH)D, 25-hydroxyvitamin D.

### Association between vitamin D level and poor sleep quality after acute ischemic stroke

The results of unadjusted (univariate analysis) and adjusted multivariate logistic regression analyses were shown in Table 3. Reduced level of 25(OH)D was associated with a significantly higher risk of poor sleep quality [odds ratio (OR) = 4.981, 95% CI = 2.320–10.691, *P*-trend < 0.001]. After adjusting for conventional factors and significant variables in the univariate analyses, including age, sex, education years, history of hypertension, history of diabetes, current drinking status, baseline NIHSS score, mRS score, and BI score, the association did not appreciably change (Model 1: OR = 6.768, 95% CI = 2.331–19.653, *P*-trend < 0.001). Furthermore, to exclude the effects of depression on the quality of sleep, we further adjusted for the covariates from Model 1 and the HAMD score; the association of low 25(OH)D with poor sleep quality was not attenuated (Model 2: OR = 6.199, 95% CI = 2.066–18.600, *P*-trend < 0.001).

**TABLE 3:** Multivariate adjusted odds ratios for the association between serum 25(OH)D levels and poor sleep quality after stroke.

	OR (95% CI)*^a^*				*P*-value for trend*^b^*	Decrease per 1 nmol/L
			
	Quartile 1	Quartile 2	Quartile 3	Quartile 4		OR (95% CI)
Unadjusted	4.981 (2.320–10.691)	2.786 (1.278–6.072)	1.205 (0.516–2.812)	1.000 (reference)	<0.001	1.031 (1.019–1.044)
*P*-value	<0.001	0.010	0.666			<0.001
Model 1*^c^*	6.768 (2.331–19.653)	5.097 (1.757–14.787)	0.992 (0.294–3.346)	1.000 (reference)	<0.001	1.040 (1.023–1.057)
*P*-value	<0.001	0.003	0.989			<0.001
Model 2*^d^*	6.199 (2.066–18.600)	5.076 (1.678–15.354)	1.041 (0.298–3.631)	1.000 (reference)	<0.001	1.040 (1.022–1.058)
*P*-value	0.001	0.004	0.950			<0.001
Model 1*^c^* (HAMD > 7)	4.264 (1.093–16.642)	5.532 (1.350–22.665)	1.191 (0.247–5.754)	1.000 (reference)	0.004	1.041 (1.015–1.066)
*P*-value	0.037	0.017	0.827			0.001
Model 1*^c^* (HAMD ≤ 7)	8.174 (2.432–27.473)	2.955 (0.886–10.084)	2.266 (0.687–7.473)	1.000 (reference)	0.005	1.043 (1.014–1.074)
*P*-value	0.001	0.084	0.179			0.004

OR, odds radio; CI, confidence level.

^a^Reference OR (1.000) is the Quartile 4 of 25(OH)D.

^b^Test for trend based on variable containing median value for each quartile.

^c^Model 1: adjusted for age, sex, education years, current drinking status, history of diabetes, history of hypertension, baseline of NIHSS scores, mRS score, and Barthel Index score.

^d^Model 2: adjusted for covariates from Model 2 and further adjusted for HAMD score.

In this study, 99 participants (33.2%) were diagnosed with depression at the 1-month follow-up. An interaction analysis found that the HAMD score influenced the effects of the 25(OH)D quartiles on sleep quality (*P* < 0.001). That is, there was a significant interaction between the effects of serum 25(OH)D and depression status on sleep quality. Therefore, we further stratified the patients by depression. Consequently, low levels of 25(OH)D were still significantly associated with poor sleep quality in both depressed patients (OR = 4.264; 95% CI, 1.093–16.642; *P*-trend = 0.004) and patients without depression (OR = 8.174; 95% CI, 2.432–27.473; *P*-trend = 0.005) (Table 3).

**FIGURE 4:**
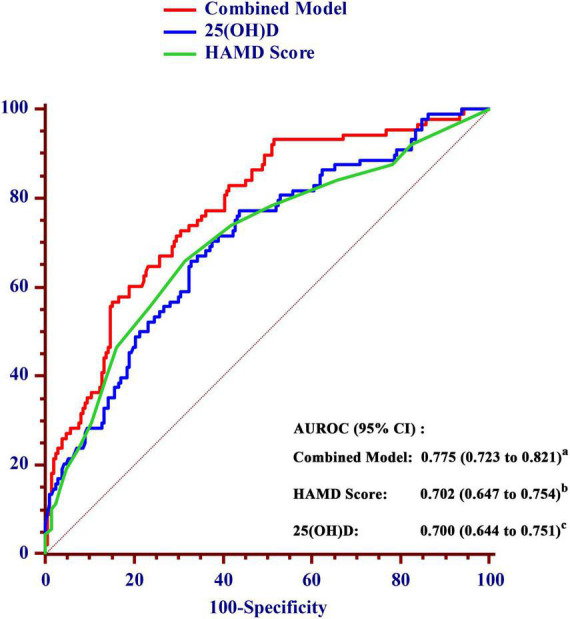
Comparison of the area under the receiver operating characteristic curve (AUROC) for prediction of poor sleep quality within 1 month between the value of 25(OH)D and the combined model in AIS patients. a vs. b, *P* = 0.012; a vs. c, *P* = 0.002. AUROC, area under the receiver operating characteristic curve; CI, confidence interval; HAMD, Hamilton Depression Scale; 25(OH)D, 25-hydroxyvitamin D.

Receiver operating characteristic curve analysis was used to evaluate the diagnostic accuracy of the serum 25(OH)D level for predicting the risk of poor sleep quality at 1 month after stroke onset (Figure 4). Considering the significant impact of depression on sleep quality, we combined the HAMD score and 25(OH)D to construct a combined model. The AUROC of the combined model for poor sleep quality at 1 month was 0.775 (95% CI, 0.723–0.821), which was significantly higher than the AUROC of HAMD score (0.702; 95% CI, 0.647–0.754) and 25(OH)D alone (0.700; 95% CI, 0.644–0.751).

## Discussion

The main finding of our work was that low serum levels of vitamin D was significantly associated with poor sleep quality at 1 month after AIS. To the best of our knowledge, there are rare studies explored the possible association between serum vitamin D levels and the development of sleep quality in stroke patients. In addition, our findings revealed the combination of vitamin D value and HAMD score showed high diagnostic accuracy in sleep quality among stroke patients.

Indeed, a total of 29.6% of AIS patients presented with poor sleep quality at 1 month in this study, which was in agreement with the results of recent studies (30, 31). Since we excluded critical patients who could not cooperate, the incidence of poor sleep quality was slightly lower. The results showed that the NIHSS and mRS scores were significantly higher among the stroke patients with poor sleep quality than in those with good sleep quality. The NIHSS and mRS scores reflect the severity of stroke. Our results indicated that sleep quality was worse in patients with severe neurological deficits, suggesting that the occurrence of sleep disorders was closely related to the extent of the neurological deficits. This was similar to previous studies (32–34). A possible reason is that limb movement disorders are common among stroke patients. Some stroke patients develop myotonia and muscle tension which may lead to inconvenient movements, nocturnal urination, and difficulty in coughing up phlegm and getting out of bed, which all affect sleep quality (35, 36).

At present, serum 25(OH)D level <50 nmol/L was defined as vitamin D deficiency (37, 38). In our study, the value of serum 25(OH)D in Q1 (3.00–30.57 nmol/L) and Q2 (30.81–48.20 nmol/L) were both less than 50 nmol/L, which could be considered as vitamin D deficiency. We found that the lowest (Q1) and second-lowest level (Q2) of 25(OH)D were both significantly associated with poor sleep quality in all multivariate-adjusted logistic regression models. These results were consistent with the previous study which demonstrated that lower level of serum 25(OH)D was associated with shorter sleep duration and lower sleep efficiency in the community cohort (39). The main reason for the lack of risk of poor sleep quality in third-lowest level (Q3) of 25(OH)D may be that vitamin D in Q3 (48.72–65.11 nmol/L) could only be considered as insufficient, and normal physiological needs could be maintained by body compensation (40, 41). In this study, we focused on the effects of deficiency and severe deficiency of vitamin D on sleep quality, and we found that the degree of 25(OH)D deficiency is negatively correlated with the quality of sleep in AIS patients.

Vitamin D, known as a neurosteroid hormone, may play an important role in the sleep process. However, the exact mechanism and causal relationship between serum vitamin D levels and sleep quality in stroke patients are still unclear. In some animal studies, investigators found that the vitamin D hormone-target neurons are broadly presented in many areas of the brain and spinal cord, some of which are considered to be involved in the pathophysiology of sleep disorder, including anterior and posterior hypothalamus, substantia nigra, midbrain central gray, raphe nuclei, and the nucleus reticularis pontis oralis and caudalis (42, 43). Similarly, receptors for vitamin D and the vitamin D catalytic enzymes were also found in the same regions of the brainstem and hypothalamus in a study of immunohistochemical investigations (44). What is more, the regulation of vitamin D on the immune system may also contribute to the relationship between sleep and inflammatory response (45, 46). In a word, serum vitamin D levels may play a critical role in the development of sleep quality through various mechanisms.

Our results also demonstrated that depression was a risk factor for the development of sleep disorders after stroke, which was consistent with the findings of previous studies (1, 10). Furthermore, we found that there was significant interaction between vitamin D levels and HAMD score in stroke patients. A recent review summarized depression is common in sleep disorders patients (47), and Buysse et Al. also confirmed that sleep disorders and depression may interact with each other (48). Therefore, we combined vitamin D levels with HAMD score to further predict sleep quality in AIS patients and gained greater confidence in discriminating post-stroke sleep quality.

Our study had several limitations. First, we excluded patients with severe aphasia or serious dysarthria, which may cause some bias on the selection of objects and affect the results. Second, serum 25(OH)D levels were examined only once at admission, further researches are needed to assess how serum25(OH)D levels change across time before and after stroke. Third, our follow-up period was 1 month, which might be too short for us to explore the long-term effects of vitamin D on post-stroke sleep quality. Three- or six-months’ follow-up are needed in further researches.

## Conclusion

In conclusion, our findings demonstrated a strong relationship between lower serum vitamin D levels at admission and the risk of poor sleep quality at the 1 month after stroke in Chinese. Combining vitamin D and HAMD score could provide a better diagnostic accuracy in discriminating poor sleep quality, and we recommended that clinical staff should pay attention to both parameters in order to select the most appropriate therapy for their patients.

## Data availability statement

The original contributions presented in this study are included in the article/supplementary material, further inquiries can be directed to the corresponding authors.

## Ethics statement

The studies involving human participants were reviewed and approved by the Ethics Committee of the First Affiliated Hospital of Wenzhou Medical University. The patients/participants provided their written informed consent to participate in this study.

## Author contributions

GH, BZ, and JH conceived and designed the project. GH, JC, YL, and JL collected the data. GH and LZ conducted the data analysis. GH draft the manuscript. All authors have made an intellectual contribution to the manuscript and approved the submission.

## Conflict of interest

The authors declare that the research was conducted in the absence of any commercial or financial relationships that could be construed as a potential conflict of interest.

## Publisher’s note

All claims expressed in this article are solely those of the authors and do not necessarily represent those of their affiliated organizations, or those of the publisher, the editors and the reviewers. Any product that may be evaluated in this article, or claim that may be made by its manufacturer, is not guaranteed or endorsed by the publisher.
